# Perspective of Pakistani Physicians towards Hospital Antimicrobial Stewardship Programs: A Multisite Exploratory Qualitative Study

**DOI:** 10.3390/ijerph16091565

**Published:** 2019-05-05

**Authors:** Khezar Hayat, Meagen Rosenthal, Ali Hassan Gillani, Panpan Zhai, Muhammad Majid Aziz, Wenjing Ji, Jie Chang, Hao Hu, Yu Fang

**Affiliations:** 1 Department of Pharmacy Administration and Clinical Pharmacy, School of Pharmacy, Xi’an Jiaotong University, Xi’an 710061, China; khezar.hayat@uvas.edu.pk (K.H.); hassangillaniali@yahoo.com (A.H.G.); caitlinzhai@163.com (P.Z.); pharmajid82@yahoo.com (M.M.A.); yfyx_8312@163.com (W.J.); jiechang@mail.xjtu.edu.cn (J.C.); 2 Center for Drug Safety and Policy Research, Xi’an Jiaotong University, Xi’an 710061, China; 3 Shaanxi Centre for Health Reform and Development Research, Xi’an 710061, China; 4 Institute of Pharmaceutical Sciences, University of Veterinary and Animal Sciences, Lahore 54000, Pakistan; 5 Department of Pharmacy Administration, School of Pharmacy, University of Mississippi, Oxford, MS 38677, USA; mmrosent@olemiss.edu; 6 State Key Laboratory of Quality Research in Chinese Medicine, Institute of Chinese Medical Sciences, University of Macau, Macau 999078, China; haohu@um.edu.mo

**Keywords:** qualitative, physicians, antimicrobial stewardship program, antibiotic resistance, Pakistan

## Abstract

*Background:* Antimicrobial resistance (AMR) is a global threat and the antimicrobial stewardship program (ASP) is a globally used tool to combat AMR. There is little information on the views among Pakistani physicians regarding AMR and the benefits of hospital antimicrobial stewardship implementation. This study was designed to explore the physicians’ views about ASP. *Methods:* Qualitative face-to-face and telephonic interviews were conducted by using purposive sampling method with 22 physicians working in seven tertiary care public hospitals of Punjab, Pakistan. All interviews were audio recorded and transcribed verbatim. Qualitative software was used, and a thematic analysis was conducted. *Results:* Three broad themes were identified: (1) the growing concern of antimicrobial resistance in Pakistan, (2) the role(s) of healthcare professionals in antibiotic prescribing, and (3) managing antibiotic resistance in hospitals. Inadequate resources, poor healthcare facilities, and insufficiently trained medical staff were the major hurdles in ASP implementation in Pakistan. *Conclusions:* Our study found a poor familiarity of hospital ASP among physicians working in public sector tertiary care teaching hospitals, and a number of distinct themes emerged during this study that could be helpful in establishing the concept of hospital ASP in Pakistan. Overall, physicians showed a positive attitude towards the enforcement of ASP in all healthcare settings, including teaching hospitals.

## 1. Introduction

Antimicrobial resistance (AMR) is a growing global challenge [1]. It is estimated that between 700,000 to several million people die as a consequence of antimicrobial-resistant infections every year [2,3]. If it remains unchecked, AMR is predicted to be a leading cause of death worldwide, with an estimated economic burden of $100 trillion US dollars by 2050 [4]. Inappropriate use of antibiotics is regarded as one of the key drivers of the emergence of AMR [5,6]. For example, in Australia, it was determined that 47% of antibiotic use was not in compliance with patient’s microbiological reports or antimicrobial guidelines [7]. Surprisingly, 50% of antibiotic use in US hospitals was deemed unnecessary in all age groups [8].

Being a global issue, AMR is also increasing in Pakistan at an alarming rate. The major reasons of antibiotic resistance include unjustified use of antibiotics [9], sale of antibiotics without a prescription [10], improper diagnostic facilities, lack of surveillance systems, and use of antibiotics in animals [11]. The presence of unlicensed medical practitioners (a person who pretends to be a medical practitioner but lacks formal education, skill, knowledge, and training), scanty dispensing practices, and untrained healthcare professionals are also other triggering factors toward antibiotic resistance [12,13,14]. Additionally, there has been a reported 65% rise in consumption of antibiotics in Pakistan from 2000 to 2015, making the problem of AMR more severe day by day [15]. 

The implementation of an antimicrobial stewardship program (ASP) in both hospital and community settings is one of the key solutions to addressing the challenge of AMR [16,17,18,19]. ASP is a coordinated effort that promotes the judicious use of antimicrobials whilst limiting the impact of AMR [20]. The major stakeholders of ASPs include infectious disease (ID) physicians [21], clinical pharmacists [22,23], infection control practitioners [24], and nurses [25] who are playing their defined and expanded roles in preventing and treating infectious diseases. Numerous countries have implemented ASPs in different healthcare settings to get optimal health benefits in terms of reduction in AMR as well as cost-effectiveness in drug therapy [26,27,28,29,30].

In Pakistan, efforts to combat AMR were initiated by the Medical Microbiology and Infectious Diseases Society of Pakistan (MMIDSP) in 2014 in order to develop and implement indigenous ASPs throughout the country [31]. Unfortunately, not much work has been done to date to implement ASPs in public hospitals in Pakistan. To help implement ASPs in this setting, there is a need to determine the views among Pakistani physicians regarding AMR and the benefits of hospital ASP implementation. The role of physicians in terms of clinical support for ASP is well evident in literature and their perceptions vary from one institution to another [5,32,33,34]. Therefore, the current study was aimed to investigate these views among physicians working in several public sector tertiary care hospitals in Punjab, Pakistan. 

## 2. Materials and Methods

### 2.1. Study Design

This was an explorative and qualitative study design that was based on face-to-face and telephonic in-depth interviews with physicians to determine their views about ASP. Semi-structured interviews are useful when collecting attitudinal information or when the research is exploratory. This design has several advantages, including its flexibility and ability to provide an in-depth investigation of the attitudes, experiences, and intentions of participants [35]. Furthermore, the interviewer also has the freedom to probe the participants to elaborate on the original response or to follow a line of inquiry introduced by the participant [36]. 

### 2.2. Study Setting

The study was conducted in three cities (Lahore, Multan, and Sargodha) of Punjab, which is the most populous province of Pakistan [37]. These cities have different social economic development levels, with Lahore having the highest gross domestic product (GDP) per capita (GDP is one of the primary criteria used to determine the economic health of a country) and Sargodha having the lowest. Lahore is the capital city of Punjab and the second largest city of Pakistan, with a population size of 11,126,285 [38]. Most of the patients residing in Punjab visit Lahore for their treatment, as it has a well-developed healthcare system, with seventeen public and forty-nine private hospitals [39]. It is estimated that more than 70,000 patients visit the outpatient department (OPD) and 30,000 patients visit emergency department of different hospitals in Lahore every day [6]. Multan is the seventh largest city of Pakistan, located in the south region of Punjab, with 1,871,843 inhabitants [38]. Sargodha is the 12th largest city of Pakistan and is situated in the northwest part of Lahore, having 659,862 residents [38].

### 2.3. Interview Guide Development

A semi-structured interview guide was developed after a thorough literature survey [33,40,41,42,43,44,45,46]. The literature survey was conducted in order to develop research question(s), to answer the research problem, and to address gaps in the existing literature. Similar to survey construction, putting together an interview protocol demands careful consideration of what information is being sought from the interviewee(s) and how this information can be extracted. We used ScienceDirect, PubMed, Google Scholar, and Web of Science to search for articles related to antimicrobial resistance and the antimicrobial stewardship program and extracted the desired information as per our study question. The reliability and validation of the interview guide was done prior to the start of the study. Two qualitative experts with relevant backgrounds reviewed and validated the guide. The reliability was assured by face-to-face interviews with the participants. The pilot study was conducted on two potential interviewees (who were not included in the final analysis), with researchers going over the questions with them to check for their understanding of vocabulary, for the relevance and importance of questions, and for their ability to answer the questions from their own knowledge and experience. The participants were able to understand the terms that were used in the interview guide, questions were meaningful, and they thought the questions were relevant to their experience and/or were effective in tapping what they knew. Minor modifications were made as per their feedback. The guide was comprised of two segments. The first part focused on antimicrobial use and its resistance, whereas the second part assessed ASP knowledge, attitudes, and barriers in the implementation of ASP in public sector hospitals of Pakistan (see Appendix A for interview guide). 

### 2.4. Participant Enrollment 

Participants included physicians who prescribe antimicrobials, were purposively sampled from seven tertiary care public teaching hospitals. A snowball sampling strategy to recruit participants was used [47]. Enrolled participants were requested to ask their fellow physicians to participate in the study. Physicians were invited to participate by telephone. Once they consented, face-to-face interviews were conducted by a trained investigator (KH) between January and March 2018. However, two interviews were conducted telephonically.

All interviews were conducted in English, since physicians were fluent in English (English is the exclusive language of instruction in medical colleges and universities in Pakistan). The duration of an interview ranged between 30 to 45 min. Refreshments were provided, but no other incentives were given for participation. Participation was voluntary, and all the participants were informed about their right to withdraw from study at any time. The identities of the participants were kept confidential from hospital employers and supervisors. In this way, participants had the opportunity to freely express their views. 

### 2.5. Inclusion and Exclusion Criteria

Physicians were included in this study only if they dealt with patients with infectious diseases and worked in public sector tertiary hospitals. Physicians working in private hospitals were excluded.

### 2.6. Qualitative Analysis

**Figure 1:**
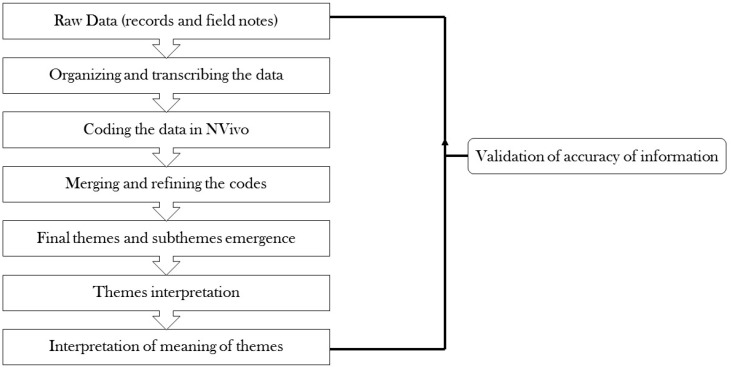
Steps used in qualitative analysis.

All interviews were audio-recorded, transcribed verbatim, and de-identified. The major themes and sub-themes were identified by the research team after thematic content analysis by utilizing steps previously described elsewhere [48,49,50]. Two researchers (KH and MR) coded the interviews using qualitative data management software (NVivo version 12 Plus, QSR International, Melbourne, Australia). After all coding was completed, a comparison was made across the coded interviews to ensure agreement and then coding was finalized through consensus (Figure 1). Saturation was achieved at the 18th interview, however, four extra interviews were conducted to confirm the saturation level. The point after which no theme and concept emerges is called a saturation point [51,52]. A continuous interaction was made with all the participants to ensure the credibility of the research.

### 2.7. Ethical Permission

Ethics approval was obtained from Biomedical Ethics Committee of Xi’an Jiaotong University (no. 2018-45). The study was also approved by the University of Veterinary and Animal Sciences, Lahore, Pakistan. Written and verbal consent was taken from all enrolled participants prior to the interviews. 

## 3. Results 

In total, 22 interviews were conducted. Seventeen participants were male. Most (59.09%) of the participants were between 20–40 years of age. Seven participants were split between 16–20 years of practice (Table 1).

**Figure 2:**
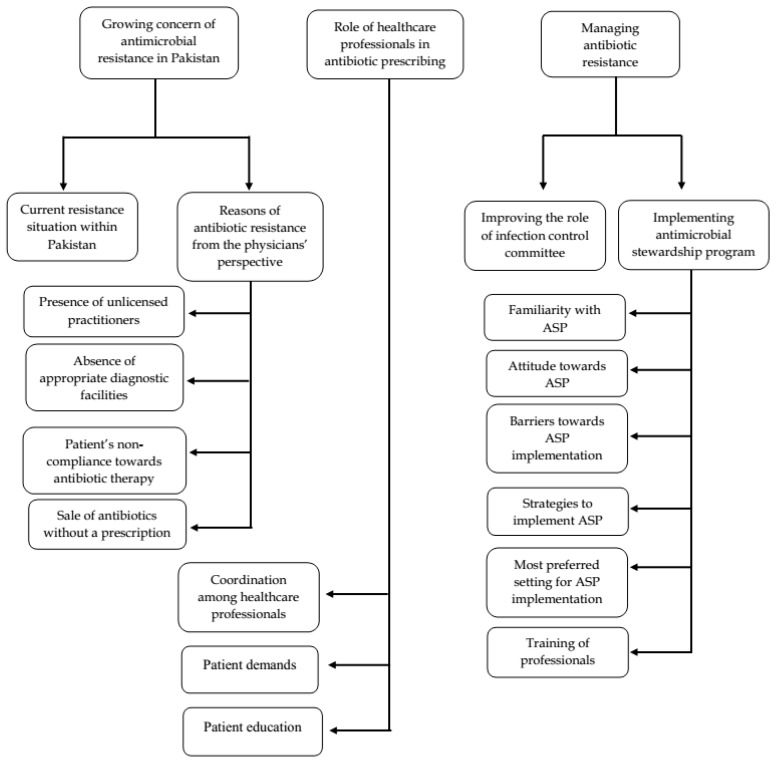
Summary of emerged themes and sub-themes.

The themes that emerged from the semi-structured interviews were broadly classified into three major categories: (1) growing concern of antimicrobial resistance in Pakistan, (2) the role of healthcare professionals in antibiotic prescribing, and (3) managing antibiotic resistance (see Figure 2).

### 3.1. Growing Concern of Antimicrobial Resistance in Pakistan 

#### 3.1.1. Current Resistance Situation 

Questions related to AMR were asked of all the participants. Most of them were of the view that antibiotic resistance is escalating gradually in Pakistan and rational approaches are urgently needed to help tackle it.

“Resistance of course is increasing in Pakistan and, unfortunately, we are facing this problem with higher intensity every year (...) Now, at this stage I feel that a simple sore throat infection that was easily cured by erythromycin is no longer cured due to the emergence of resistance.” (P7)

“There is no doubt that there is a strong relationship between irrational use of antibiotics with antibiotic resistance and it is continuously growing in Pakistan.” (P2)

#### 3.1.2. Reasons of Antibiotic Resistance from the Perspective of Physicians 

The reasons of antibiotic resistance were well explained by the participants. 

##### Presence of Unlicensed Medical Practitioners

The participants revealed the presence of unlicensed practitioners as a major problem towards the increasing rate of antibiotic resistance due to their unjustified medical practice.

“Unfortunately, there are (a) number of other so-called doctors such as unlicensed practitioners who most of the times prescribe antibiotics in an irrational way, thus increasing the risk of antibiotic resistance.” (P6)

“In our country, unlicensed practitioners prescribe antibiotics without any rational approach and (are) amplifying the problem of antibiotic resistance.” (P16)

##### Absence of Appropriate Diagnostic Facilities

Some physicians said that the absence of standard diagnostic facilities contribute toward the irrational prescribing of antibiotics.

“There is a lack of appropriate diagnostic facilities, especially in basic health units and district hospitals. This further promotes the use of antibiotics without culture sensitivity tests.” (P8)

“Physicians in the periphery and remote areas mostly use broad spectrum antibiotics and they do not bother to recommend lab tests, maybe due to (the) absence of diagnostic facilities.” (P5)

##### Patient’s Non-Compliance towards Antibiotic Therapy

The participants suggested the need of adequate patient education about antibiotic use and ways to reduce or prevent antibiotic resistance.

“Non-compliance with antibiotic treatment is common among patients, as most of them don’t complete their treatment course and when they use the same medicine in future, it is unable to cure them due to the emergence of resistance.” (P9)

“They (patients) will stop their antibiotics once they think that they are feeling better and do not complete the recommended duration of therapy as we have instructed them.” (P12)

“Resistance against antibiotics is increasing just like (in) other countries (….) there is a need to educate and encourage patients to complete their antibiotic course to prevent the risk of resistance against antibiotics.” (P21)

##### Sale of Antibiotics without a Prescription

The participants recommended the enforcement of strict laws to limit the purchase of antibiotics without a prescription in order to cut the risk of antibiotic resistance.

“Unfortunately, self-medication is very common, and you can take any drug like antibiotics freely from the medical store or pharmacy without a prescription.” (P5)

“In my view, the origin of resistance is the inappropriate use of antibiotics by the community. This risk will be higher if they are able to get these pills without any prescription.” (P11)

“I think the government should take meaningful steps to prevent the sale of antibiotics without a prescription.” (15)

### 3.2. Role of Healthcare Professionals in Antibiotic Prescribing

#### 3.2.1. Coordination among Healthcare Professionals

Participants highlighted the need for coordination among different healthcare professionals, including physicians, nurses, microbiologists, and pharmacists, to help improve antibiotic prescribing and use in hospital patients.

“A major weakness in government sector hospitals is (the) lack of mutual cooperation among different healthcare professionals, which could affect the rational use of antibiotics in hospitals.” (P3)

“Antibiotic prescribing is a team (effort) in which every professional is doing his/her job (……) being a physician, we can discuss about antibiotic therapy with pharmacists and nurses. Obviously, microbiologist will be helpful for lab results. So, in this way, we will have evidence-based antibiotic therapy, which will improve the quality of life of patients, but unfortunately this coordination is missing.” (P13)

“No doubt if all the stakeholders of our healthcare system including physicians, pharmacists, nurses, and microbiologists work in coordination then it will definitely improve antibiotic usage within a hospital.” (P15)

#### 3.2.2. Demand of Antibiotics 

Some physicians also explained that patients tried to influence them by demanding antibiotics as part of their drug therapy despite the nature of their infection.

“[Patients] sometimes demand antibiotics and try to influence our clinical judgment, especially in government hospitals. They know basic information about antibiotics, like names of certain very common antibiotics, which (were) possibly tracked from friends or the Internet, and they are pre-occupied with receiving a specific antibiotic. So, they even ask us to prescribe that antibiotic.” (P3)

“This is a demand of patients to be given an antibiotic, but we try to prescribe only if it is suitable for them. Some patients come after evaluation from several doctors and show us their previous prescriptions and tell us which antibiotic showed better results, then in such situations, we sometimes prescribe the same antibiotic.” (P6)

#### 3.2.3. Patient Education 

Participants were asked about how they educate patients if they ask for antibiotics for their viral infections.

“Well, there are certain infections like flu in which antibiotics should not be given. I try to counsel such patients and tell them that it’s nothing but an extra expense from your pocket. Most of the times, patients agree to what I say.” (P1)

“We educate patients suffering from viral infections by saying that symptomatic relief is all that you need, and antibiotics use will be ineffective in this case. In reality, self-medication with antibiotics is very common in our community and the rate of compliance is higher if you prescribe a greater number of pills to the patients regardless of their clinical needs.” (P7)

### 3.3. Managing Antibiotic Resistance

#### 3.3.1. Improving the Role of Infection Control Committee

Most of the participants were not happy with the role of infection control committees in their hospitals and suggested that its role should be enhanced to curtail antibiotic resistance.

“Yes, we do have an infection control committee in our hospital, but at the moment I am not satisfied with its current performance due to [the] lack of its active role in reducing the risk of hospital-acquired infections as well in the development of infection prevention policies and treatment guidelines. Its performance must be improved to address resistance issues.” (P20)

“The infection control committee exists in our hospital, but it is in a very pathetic condition. Its working is not up to the mark, probably due to insufficient funds and inadequate staff. (……) There is a need to improve its role to reduce antibiotic resistance.” (P3)

#### 3.3.2. Implementation of ASP in Pakistani hospitals

##### Familiarity

During the interview, participants were asked about their understanding of hospital ASPs. Only a handful of physicians had some knowledge about an ASP. Detailed written information was provided to all participants to elicit their views on hospital ASPs.

“To be honest, I am not aware of this program, but now I have some basic information about it after a discussion with you. It seems to be a new concept to rationalize the use of antimicrobials including antibiotics.” (P14)

“Unfortunately, I do not have any prior information about this program.” (P13)

##### Attitude towards ASP

All participants interviewed showed a positive attitude towards implementation of ASP in Pakistani hospitals.

“I personally do not have any reservation in (regard to) its (ASP) implementation, as it is not only useful for patients but also for physicians.” (P11)

“It is a very useful program and, being a physician, I have (a) very positive attitude towards ASP due to its benefits in (terms of reducing) antibiotic resistance, which will speed up the prognosis rate among patients. If we consider ASP in ideal condition(s) then, of course, this system will have multiple merits.” (P9)

##### Barriers towards Implementation of ASP

Participants indicated several barriers in ASP implementation in Pakistani hospitals, but all those barriers could be overcome if government provides necessary support, including financing.

“There will be a lot of barriers. For example, we have heavy patient loads and it would be too difficult for us to get a prior approval of an antibiotic from an ID physician every time (…) Secondly, there will be a need of huge financing to implement hospital ASPs.” (P8)

“I perceive that budgeting, which is the main hindrance, and inadequate resources could be the major barriers in its implementation.” (P1)

“We may not have enough qualified and trained professionals like ID physicians, nurses, microbiologists, and clinical pharmacists who have experience of working in (an) ASP healthcare facility.” (P17)

##### Preferred ASP Strategies 

There are different strategies that can be used to adopt ASPs in hospitals, such as prior approval from ID physician/formulary restriction, and prospective audit with feedback. There were mixed views from participants when the question related to a preferred strategy for hospital ASP implementation was asked.

“As per my opinion, the second one [formulary restriction] will be more effective, as this will restrict the use of antimicrobials. This restriction should be specific to every hospital, but some physicians may resist in order to seek approval before prescribing antibiotics.” (P7)

“Prior approval of prescribing antibiotics will be more beneficial in Pakistan, as this will act as a check point and limit the prescribing of antibiotics by physicians, but this may also challenge the authority of the physicians.” (P4)

“I think we should try to implement a combination of different ASP strategies to determine the best suitable and useful option.” (P12)

##### Preferred Hospital Setting for ASP Implementation

In Pakistan, there are different types of hospital settings, including tertiary care hospitals and secondary care hospitals. During the interviews, participants were asked about the type of hospital settings where ASP should be preferably implemented.

“Well, we must start this program from tertiary healthcare units and district hospitals, where there are more trained staff and experts. Once they understand it, then they will teach to their juniors and (knowledge will) trickle down from top to bottom.” (P1)

“I think ASP should be implemented first in tertiary hospitals and then step by step to other hospital settings, including secondary and primary.” (P10)

##### Training of Healthcare Professionals 

All participating considered ASP training as an important step before its adoption.

“Yes, obviously, training is needed in order to get better understanding in the form of workshops or conferences and I think healthcare professionals should have (a) certification from an approved course in order to be the part of ASP.” (P5)

“It will be a good option to train the physicians before the implementation of this program. We need to do some pilot project first and if results are positive then we can continue it with zeal.” (P10)

“Well, training is always necessary in order to get better results. So, all healthcare professionals should be trained before the implementation of ASP in hospitals. In my view, the government should plan a training program accompanied with a certificate, so that only a trained healthcare professional could join the ASP team.” (P18)

## 4. Discussion

The major themes of this study are antibiotic resistance as a problem in Pakistan, the role of healthcare professionals in antibiotic prescribing, and managing antibiotic resistance by the implementation of hospital ASPs in Pakistan. The present study has highlighted good familiarity among most of the physicians towards the antibiotic resistance problem in Pakistan. They were aware that this problem is gaining momentum and that rigorous efforts are needed to cope with this issue. The participants revealed some major factors for the prevalence of resistant microbes in Pakistan, including the sale of antibiotics without a prescription, as well as the presences of unlicensed medical practitioners, self-medication, and poor diagnostic facilities. These findings are similar to those in prior published studies [10,16,53,54,55]. The Punjab Healthcare Commission (PHC) is taking serious measures, including the closure of clinics of unlicensed practitioners to prevent their unjustified medical practices [56]. 

In this study, most of the participants were not satisfied with the performance of their infection control committee. So, there appears to be a need to strengthen the functions of infection control committees in hospitals, as this will help in the prevention of the nosocomial spread of infections and resistant pathogens. In the absence of control measures, hospitals may act as a reservoir of antibiotic resistant strains that could be disseminated to the community [1,11,57]. 

ASP is still a new approach in Pakistan to curtail the problem of antibiotic resistance. There has already been a lot of work reported in different developing countries [26,58,59], but views among Pakistani physicians towards ASP are uncertain due to a dearth of local information available on the subject. This study highlighted poor knowledge among interviewed physicians regarding ASP. As the continuing medical curriculum, both at undergraduate and post-graduate levels, does not have any topic related to ASP [60], it is perhaps unsurprising that physicians are currently not aware about the functions of a hospital ASP. The views of participants towards the implementation of ASP in public sector hospitals of Pakistan showed readiness to accept the change. Furthermore, the implementation of hospital ASPs in Pakistan will be challenging, but it is a need of the hour, as many countries have successfully implemented ASP in their different hospital settings due to its merits in regard to patient care [18,23,26]. 

According to the results of this study, an ASP-certified training program would be of great benefit not only to physicians but also for nurses, microbiologists, and clinical pharmacists. All the participants agreed that training programs would improve their familiarity with objectives and core elements of ASP. Our study also found that a lack of resources in terms of both staff and infrastructure creates the major hurdles toward ASP implementation. Unfortunately, there are very few infectious disease physicians working in Pakistan, and the doctor to patient ratio is alarmingly low (1:1300) [61,62]. 

Two approaches for hospital-based ASPs are currently endorsed by national organizations and include preauthorization/formulary restriction and prospective audit with feedback [63,64]. The majority of the participants in our study were of the view that there is a need to implement an antimicrobial restriction policy in order to limit the use of antibiotics. However, they revealed that in the case of an antimicrobial restriction policy, physicians will need to seek prior approval from an ASP team to prescribe antibiotics from the control list of the hospital formulary. This may challenge the authority of the physicians, which may limit the acceptance of ASP [65,66]. Antibiotic approval systems have been shown to be useful in a range of contexts, but the capacity of such a system to enact change is dependent on the experience and ability of prescribers to use such a system. Different antibiotic approval systems such as Electronic decision-support or telephone-based approval systems are already in practice in different countries. However, the acceptability of antibiotic approval systems could be improved by incorporating resources and providing time to maintain and foster professional relationships [66]. 

## 5. Limitations

To the authors’ knowledge, this study is the first of its kind to investigate the views among Pakistani physicians regarding hospital ASPs. However, there are limitations that should be noted in this study. First, this study cannot depict a complete picture of all physicians of Pakistan, as it was conducted in only three cities of Punjab province. Having said this, considering that it took place in the most populous province of Pakistan and among experienced physicians working in different tertiary hospitals, the findings may be applicable to other parts of the country. Second, there is a potential researcher bias. To reduce individual biases, we involved several researchers with pharmacy backgrounds and knowledge of ASP concepts to assist with coding and theme development. Finally, only physicians from public sector hospitals were included, and views among physicians working in private hospitals may differ from those expressed in this study. 

## 6. Conclusions

In conclusion, a diminished understanding of hospital ASPs was found among physicians working in public sector tertiary care hospitals of Punjab, Pakistan. However, they have shown a positive attitude towards its enforcement in all healthcare settings, including teaching hospitals. Insufficient resources, poor healthcare facilities, and inadequate training of medical staff are the major hurdles in ASP implementation in Pakistan. The onus may now be on government policy to help initiate ASP in all public sector hospitals in the country.

## Figures and Tables

**Table 1:** Demographic information of participants (*n* = 22) and hospital characteristics (*n* = 7).

Characteristics	Total *n* (%)
**Age (years)**	
20–30	4 (18.18)
31–40	9 (40.91)
41–50	7 (31.82)
>50	2 (9.09)
**Gender**	
Male	17 (77.27)
Female	5 (22.73)
**Experience (years)**	
1–5	3 (13.64)
6–10	7 (31.82)
11–15	2 (9.09)
16–20	8 (36.36)
>20	2 (9.09)
**Education**	
MBBS ^1^	10 (45.45)
FCPS ^2^	12 (54.55)
**Nationality**	
Pakistani	22 (100)
**Hospital departments**	
Medicine	11 (50)
Surgery	6 (27.27)
Obstetrics & Gynecology	5 (22.73)
**Frequency of antibiotic prescribing/day**	
6–10 prescription	6 (27.27)
>10 prescriptions	16 (72.73)
**Bed capacity of hospitals (*n* = 7)**	
<1000	2 (28.57)
1000 to 1500	4 (57.14)
>1500	1 (14.29)

^1^ Bachelor of Medicine and Surgery, ^2^ Fellow of College of Physicians and Surgeons.

## Appendix A

### ***Interview Guide***

Demographic information about participants will be taken prior to the interview.

#### *Antimicrobial Prescribing and Resistance*

1. How often do you prescribe antibiotics per day?
2. How do you deal with patients who demand antibiotics for their illness?
  (a) Do you counsel them?
  (b) Do they influence your antibiotic prescribing behavior?
3. In your opinion, what is the role(s) of other healthcare professionals in your antibiotic prescribing?
  (a) How do different healthcare professionals affect your prescribing of antibiotics?
4. Could you describe the role of the infection control committee in your hospital?
  (a) Are you satisfied with its performance?
5. What do you think about antimicrobial resistance in Pakistan?
  (a) Is it reducing or increasing?
  (b) If it is increasing, then what are the reasons of the increase in resistance?

#### *Perception and Attitude towards the Antimicrobial Stewardship Program (ASP)*

1. Do you know what the antimicrobial stewardship program is?
2. In your view, how can we implement ASP in the public hospitals of Pakistan?
  (a) Should we first implement ASP in tertiary care hospitals or basic health units?
3. Could you describe the strategies that are the most useful in implementing hospital ASPs in Pakistan (formulary restriction, prospective audit with feedback, combination of these strategies)?
4. What will be the benefits of implementing hospital ASPs in Pakistan?
5. How will physicians see the implementation of hospital ASPs in Pakistani?
  (a) Will they have any sort of reservations on its implementation?
6. What are your views about the training of healthcare professionals prior to the implementation of the hospital ASPs in Pakistan?
7. In your opinion, what are the barriers in successful implementation of ASP in Pakistani hospitals (unavailability of trained staff, limited resources)?
8. Are there any other comments you would like to give about antimicrobial resistance or the antimicrobial stewardship program?
